# Temporary Team Performance and Knowledge Hiding: Mediated by Interpersonal Mistrust

**DOI:** 10.3389/fpsyg.2022.876710

**Published:** 2022-06-02

**Authors:** Yurong Miao, Na Qi, E. Liu, Pengxun Zhai

**Affiliations:** ^1^Institute of Ethnic Literature, Yunnan Academy of Social Sciences, Kunming, China; ^2^Department of Life Culture, Beijing College of Social Administration, Beijing, China; ^3^School of Philosophy and Sociology, Jilin University, Changchun, China; ^4^College of Economy and Management, Southwest Forestry University, Kunming, China; ^5^Adam Smith Business School, University of Glasgow, Glasgow, United Kingdom

**Keywords:** knowledge hiding, interpersonal distrust, temporary team performance, incentive atmosphere, passive concealment of knowledge

## Abstract

Although scholars have acknowledged that knowledge hiding is negatively with team performance, none of the extant research has revealed the relationship between knowledge hiding and the performance of temporary teams. To fill this gap, we will explore whether and how knowledge hiding influences temporary team performance. Following a literature review, the correlation between knowledge hiding, interpersonal distrust, and temporary team performance is explored, and the theoretical model between variables is constructed, along with four hypotheses. Then, a quantitative analysis is conducted through a QS (Questionnaire Survey) design on the proposed hypotheses. Specifically, test is conducted on the collected data, and then SPSS and AMOS are used to integrate and analyze the data of 102 teams. The results show that knowledge hiding and its two dimensions (active and passive hiding) have a negative impact on the work efficiency of temporary teams. Interpersonal distrust plays a mediating role between knowledge hiding and temporary team performance. The incentive atmosphere, including control atmosphere and performance atmosphere, can regulate the performance of a temporary team efficiently. Control atmosphere is favorable to improving interpersonal trust and team performance, and performance atmosphere is inverted U-shaped regulation between interpersonal distrust and temporary team performance. Based on the above conclusions, the corresponding management suggestions that encouraging members in temporary teams to share actively and confronting the passive concealment of knowledge are put forward to enhance interpersonal trust and improve the efficiency of the temporary team.

## Introduction

The 21st century marks technological and industrial innovation, under which innovation has become the trendy development of the team (Dimopoulos et al., 2017; Olsen, 2019; Nemţeanu et al., 2021). Especially, given blurred enterprise boundaries, goal-oriented team cooperation is proliferating, and one typical cooperation pattern is the formation of a temporary team (Ostermann and Schreyogg, 2014; Valentine, 2018). The temporary team refers to these teams of relative strangers assembled on demand for one-time engagements and coordinating tightly coupled and complex work (Valentine, 2018). Team-based knowledge sharing can help build a good team cooperation atmosphere, improve membership trust, and thus enhance team efficiency (Srivastava et al., 2006; Mesmer-Magnus and Dechurch, 2009; Zhang et al., 2018). However, the temporary team is often unstable, lacks mutual understanding among memberships, and knowledge hiding is common (Arduin et al., 2013; Dong et al., 2017; Oyemomi et al., 2019). Knowledge hiding is often found in project teams (Zhang and Min, 2019). Relevant literature consultation shows that knowledge hiding behavior harms knowledge sharing or knowledge innovation within the team (Han, 2017; Watson et al., 2020).

The domestic literature on knowledge hiding has pointed out its main focus through keywords, such as knowledge sharing, knowledge hiding, mediating role, and interpersonal relationship (Harrower, 2019; Pera, 2019; Nemteanu et al., 2021). Chronologically, the literature before 2015 mainly discusses the concept of knowledge hiding and the factors of knowledge hiding behavior. By comparison, the latest literature addresses the role of knowledge hiding on and between different variables (Fang, 2017; Paniagua Zambrana et al., 2017; Khalid et al., 2018). Overall, domestic research has not reached an in-depth understanding of knowledge hiding behavior, but its importance has gained recognition.

Existing literature has studied the impact of knowledge hiding on interpersonal relationships and team performance through stable teams (Černe et al., 2014; Connelly et al., 2014; Zhu et al., 2019). However, knowledge hiding is a more popular problem among temporary teams (Zhang and Min, 2019). Although scholars have acknowledged that knowledge hiding is negatively with team performance (Zhu et al., 2019; Wang et al., 2021), none of the extant research has revealed the relationship between knowledge hiding and the performance of temporary teams. To fill this gap, we will explore whether and how knowledge hiding influences temporary team performance. Distinctively, here, the relationship between knowledge hiding, interpersonal relationship, and team performance is explored through temporary teams to improve the research mechanism on the mediating effect of knowledge hiding and interpersonal distrust. Consequently, shortcomings in team management are found, and knowledge hiding within the team is avoided, thereby enhancing people’s trust and improving the overall efficiency of team members.

## Research Hypotheses and Design

### Theoretical Model Establishment

**FIGURE 1:**
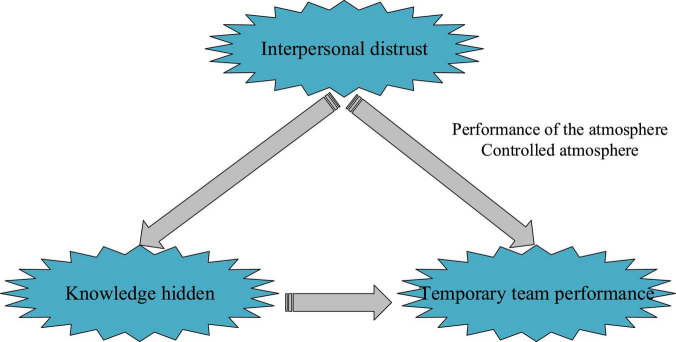
The basic theoretic framework.

The research of knowledge hiding, interpersonal mistrust, temporary team performance, and their relationships proves that knowledge hiding causes membership mistrust, thereby reducing the work efficiency of the temporary team (Travis Maynard et al., 2017; Dodokh, 2020; Rumtini et al., 2020). That is, interpersonal distrust plays a mediating role in knowledge hiding. Incentive atmosphere can be further divided into performance and control atmosphere, both of which play an important role in the relationship between interpersonal mistrust and temporary team performance (Kim et al., 2018). According to the above relationship, the theoretical model framework is established, as shown in Figure 1.

### Influence of Knowledge Hiding on Temporary Team Performance

A temporary team is a collection of people with different skills banded together for specific tasks and disbanded after tasks completion. Previous research has shown that team duration has an important impact on the team process and team performance (Chae et al., 2015). Because the team is temporary, membership distrust is extremely prominent. For better work efficiency, the membership trust must be quickly established in the shortest time, and individual knowledge and skills should be shared, which, however, is extremely difficult at the beginning because of its instability, thereby resulting in frequent knowledge hiding behaviors in the temporary team (Xiao and Cooke, 2018; Zhang and Min, 2019). Accordingly, the following hypotheses are put forward.

H1: Knowledge hiding is negatively correlated with the work efficiency of temporary teams.

Knowledge hiding, which refers the deliberate attempts by individuals to withhold or conceal their experience, task information and know-how that are required by other persons (Connelly et al., 2012; Zhang and Min, 2019). In the relevant literature, knowledge hiding is further divided into two dimensions: active hiding and passive hiding, and these two dimensions have the opposite effect on team performance. Active hiding has a negative impact on team performance, while passive hiding can improve teamwork efficiency (Dodokh, 2020; Weng et al., 2020; Wang et al., 2021). Here, it is considered that team members may have a misunderstanding of passive hiding due to mistrust, which leads to the dissatisfaction of other members, resulting in low work efficiency. Based on the above analysis, two hypotheses are put forward.

H1a: Active hiding is negatively correlated with the work efficiency of temporary team.

H1b: Passive hiding is negatively correlated with the work efficiency of temporary teams.

### The Mediating Role of Interpersonal Distrust

The cultivation of interpersonal trust is favorable to the improvement of teamwork efficiency, and knowledge hiding will lead to trust deterioration within the team. In the temporary team, members usually hide their attitude of not understanding knowledge, which will reduce their aspirations for knowledge and is not favorable to forming a good working atmosphere (Dalal et al., 2016; Valentine, 2018; Maureira et al., 2019). Passive hiding does not harm the interests of others, which will not affect members in a stable team with strong membership trust but will harm a temporary team by causing misunderstanding of passive hiding, thereby reducing the membership trust. Accordingly, three hypotheses are put forward.

H2: Interpersonal mistrust mediates the relationship between knowledge hiding and temporary team performance.

H2a: Interpersonal mistrust mediates the relationship between active hiding and temporary team performance.

H2b: Interpersonal mistrust mediates the relationship between passive hiding and temporary team performance.

### Analysis on the Regulating Effect of Incentive Atmosphere

The incentive atmosphere can be divided into two dimensions: control atmosphere and performance atmosphere. The control atmosphere encourages self-improvement. Under the control atmosphere, team members will form a good working atmosphere of helping each other. Comparatively, the performance atmosphere encourages competition among members, resulting in negative dependence among members (Zárraga and Bonache, 2005; Meng et al., 2016). Based on the above analysis and relevant literature, the following hypotheses can be put forward.

H3: The formation of control atmosphere is positively related to the cultivation of trust among team members and the improvement of work efficiency.

H4: There is an inverted U-shaped relationship between performance atmosphere and the cultivation of trust among team members and work performance.

Performance climate has an inverted U-shaped moderating effect on interpersonal trust and temporary team performance, where the inverted U-shaped moderating effect means that a small degree of performance climate will motivate team members to improve their own abilities to complete team tasks, in which case performance climate positively moderates interpersonal trust and temporary team performance, while an excessively competitive climate will negatively affect temporary team performance. In other words, performance climate negatively moderates the positive relationship between interpersonal trust and temporary team performance when the degree of performance climate is too high.

### Design and Measurement of Variables

**FIGURE 2:**
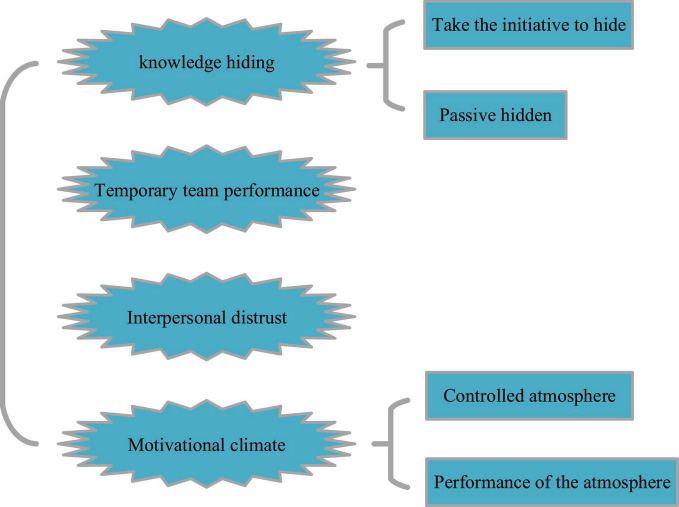
QS design framework.

Here, the samples in this paper are college teachers and students with temporary team working experience from Shanghai, Shenzhen, Guangdong, and Beijing. The QS (Questionnaire Survey) is conducted by online and offline investigation. And the cash reward for each questionnaire was 10 yuan, which encouraged the college teachers and students and obtained a high participation rate. Data were collected with confidentiality agreement to guarantee confidentiality of their responses. The QS is designed with four parts, as shown in Figure 2.

Figure 2 shows that the four parts of the QS: knowledge hiding, temporary team performance, interpersonal distrust, and incentive atmosphere, with a total of 36 questions, including 12 in the first part, 11 in the second part, 4 in the third part, and 9 in the fourth part. The QS score has cited Likert’s five-point scale, and the numbers from “completely inconsistent” to “fully consistent” are expressed by numbers 1–5, respectively (Qualter et al., 2009; Van Breukelen et al., 2012; Valentine and Edmondson, 2014; Zhu et al., 2019).

**FIGURE 3:**
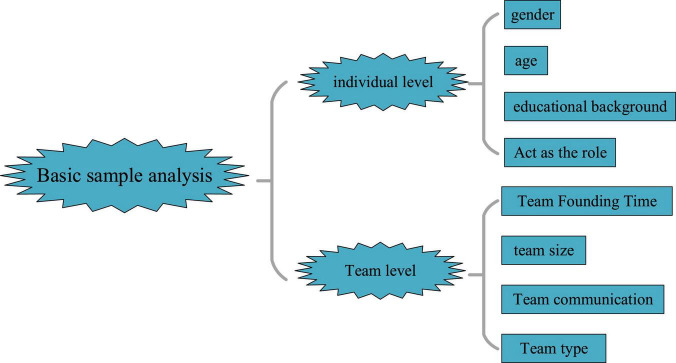
Data level in basic sample analysis.

The QS design is modified according to the maturity scale in the relevant literature. Then, the QS is distributed to 102 temporary teams in different regions in March 2020, and 463 valid QS are collected. Here, the sample analysis is involved from two levels, as shown in Figure 3.

## Empirical Analysis and Result Discussion

### Descriptive Statistics

**FIGURE 4:**
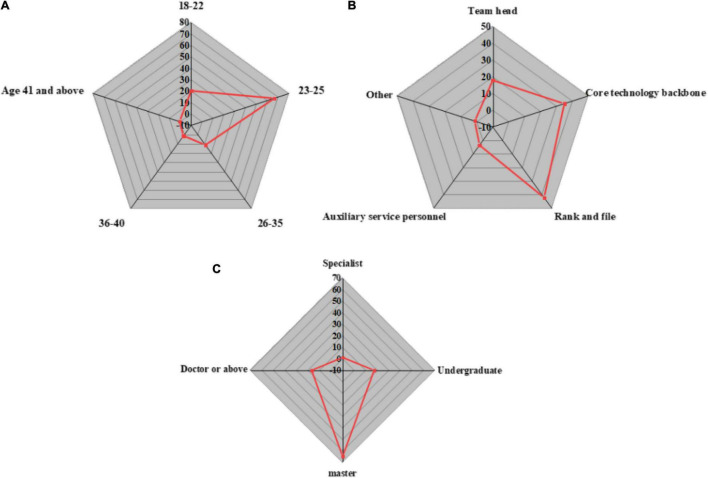
Sample basic information at the individual level. **(A)** Age; **(B)** Team role; **(C)** Education.

Statistically, boys and girls account for 52.7 and 47.3%, respectively, among the QS samples. The basic information of the individual level samples, such as their age, education level, and role in the team is shown in Figure 4:

Figure 4 indicates that people between 18 and 25 account for the most in the sample. In terms of education, the overall education level is relatively high, and the proportion of people with a master’s degree is the largest, thereby ensuring the quality of the QS. The team roles include technicians, ordinary employees, and service personnel, and the proportion of the three roles almost equals. Thus, the QS is comprehensive.

**FIGURE 5:**
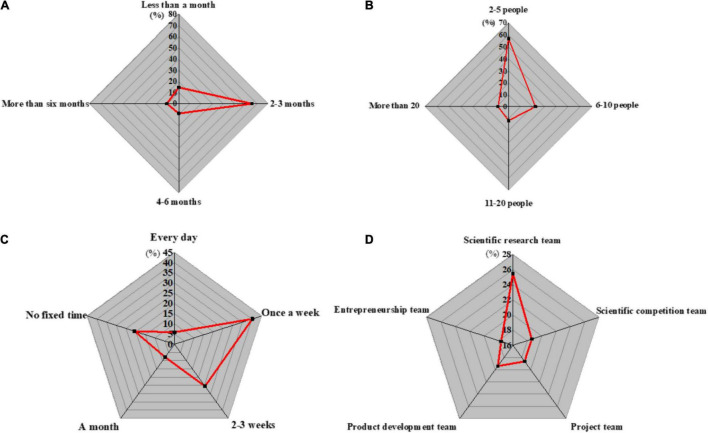
Team level sample basic information. **(A)** Team establishment time; **(B)** Team size; **(C)** Team communication; **(D)** Team type.

Further, the collected data are analyzed from the team level, and the analysis results are shown in Figure 5.

Figure 3 demonstrates that the establishment time of the team is mostly between 2 and 3 months, and the teams sized with 2–5 people account for the largest proportion. This may be to avoid the difficulty of unifying membership opinions, which reduces the overall teamwork efficiency. Most team communication time will be controlled once a week or twice a week, indicating that there is plenty of opportunities for communications. In terms of team types, the proportion of the five types of teams is basically the same, suggesting that the respondents are comprehensively considered.

### Team Level Data Analysis

(1) Rationality test of team data structure

**FIGURE 6:**
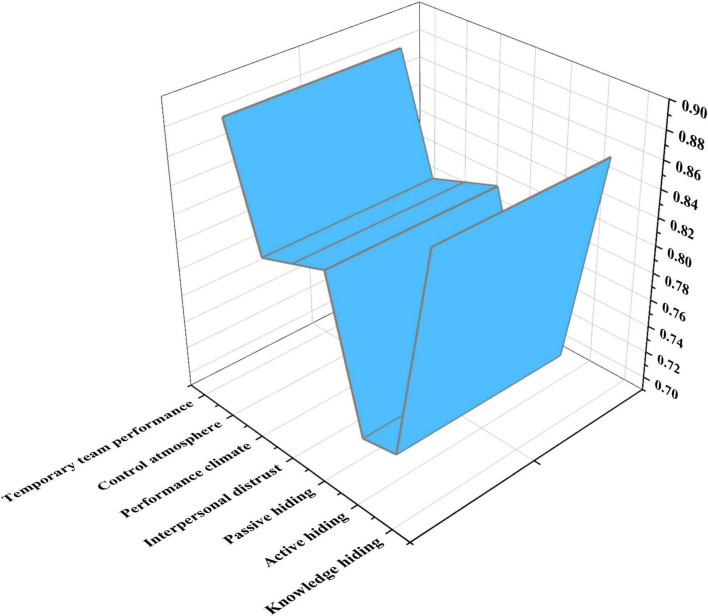
Variable’s r_wg_ coefficient of each dimension.

Here, the r_wg_ coefficient is used to judge whether the data at the individual level and team level can be integrated, which can be used to describe the consistency of the answers of team members to the QS (James et al., 1993). The QS results are analyzed using SPSS25.0 to process the obtained data and calculate the r_wg_ coefficients of each variable. The results are shown in Figure 6:

Generally, (r_wg_)_min_ > 0.7 indicates that the obtained data can be integrated. Figure 6 displays that the variable’s r_wg_ coefficient of each dimension is greater than 0.7, and there is good consistency among team members, the individual-level data can be further integrated into the team level. Hence, the data of individual members of each team are added and averaged to obtain the corresponding team-level data. Subsequently, the data obtained at the team level are used for analysis.

(2) Reliability test

**FIGURE 7:**
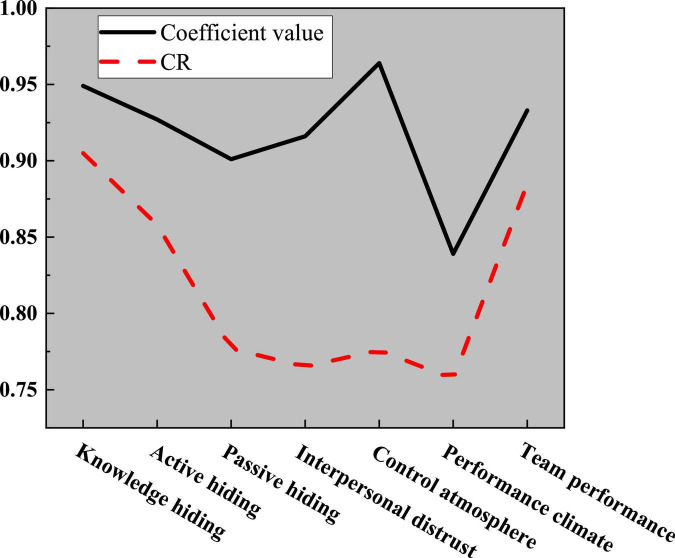
Reliability test of variables.

Here, the reliability of the collected data is tested by detecting the _α_ coefficient and CR (Composite Reliability) value of the variable’s each dimension. The results are shown in Figure 7:

Figure 7 tells that the values of variables are greater than 0.9, except for performance atmosphere, and the maximum value is as high as 0.964; CR values are greater than 0.7, indicating that the data the variable’s each dimension has high reliability, which can further test the validity of the variable.

(3) Validity test

Afterward, the variable’s each dimensional data are tested, and the Bartlett sphere test coefficient and KMO (Kaiser-Meyer-Olkin) value of the variable are calculated by PCA (Principal Component Analysis). The calculated KMO value is as high as 0.929, which is significant at Bartlett (0.000) level, and the variance interpretation rate is 69.72, which meets the relevant requirements, indicating that it is reasonable to use factor analysis to analyze the data at the team level.

**FIGURE 8:**
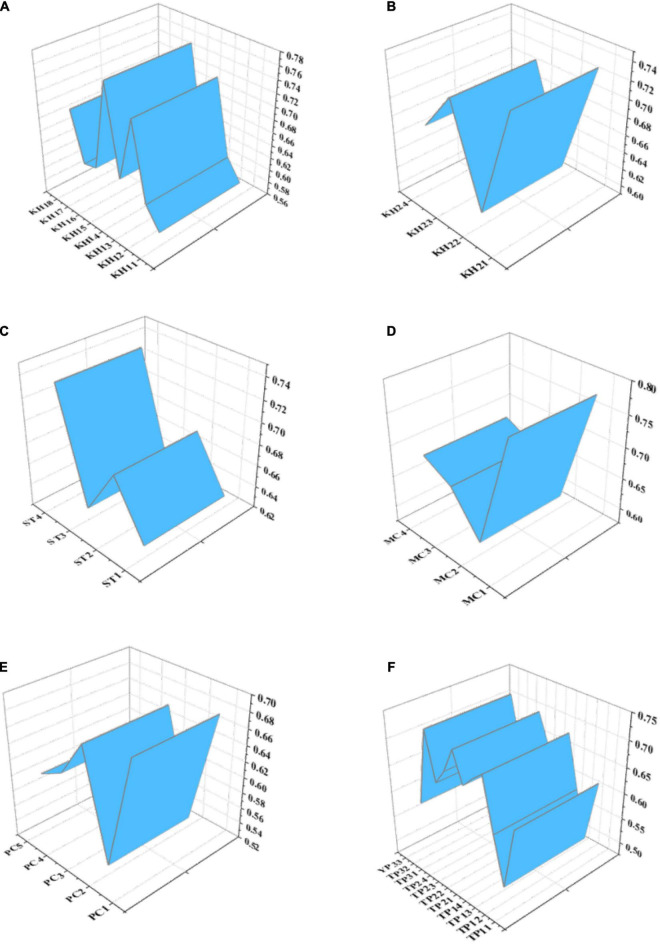
Variable factor load of each topic. **(A)** Active hiding; **(B)** Passive hiding; **(C)** Interpersonal distrust; **(D)** Control atmosphere; **(E)** Performance atmosphere; **(F)** Interim team performance.

AMOS is used to analyze the collected team-level data. The results are shown in Figure 8:

Figure 8 shows that the factor load of the questions contained in each variable is mostly above 0.6, and only a few questions have a factor load of less than 0.6. However, deleting these questions with a small factor load will lead to an incomplete QS design. Therefore, these questions with a small factor load are not deleted here.

### Regression Analysis

Subsequently, the SPSS25.0 is used to verify the proposed hypotheses, the relationship between various variables, and the interaction between various variables, and the basic information of the team is taken as the control variable to improve the analytical accuracy.

**FIGURE 9:**
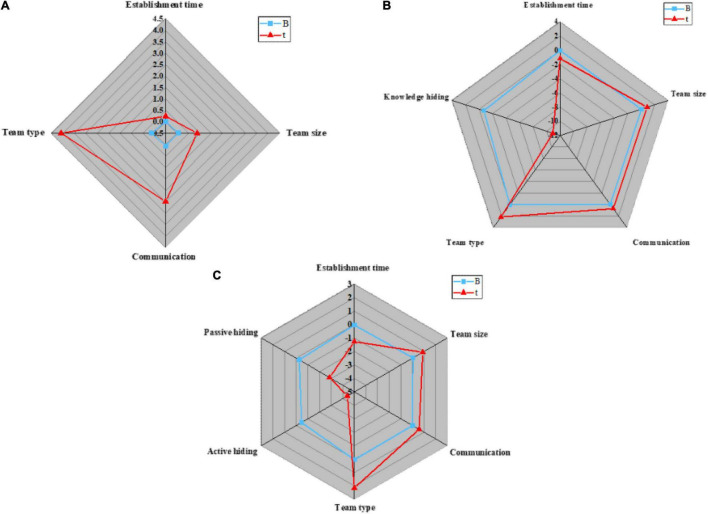
Regression analysis results of knowledge hiding on temporary team performance in different models. **(A)** Regression model 1; **(B)** Regression model 2; **(C)** Regression model 3.

**FIGURE 10:**
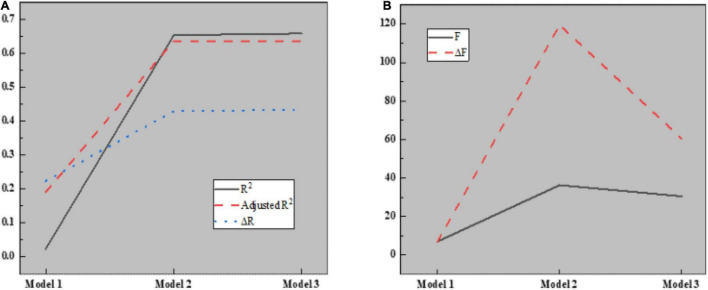
Parameters in regression analysis. **(A)** Parameters of R^2^; **(B)** Parameters of F.

(1) Regression analysis of knowledge hiding on temporary team performance

1. Hypothesis H1 verification: the control variables, such as team basic information and temporary team performance are introduced into regression model 1; the independent variable knowledge hiding and dependent variable temporary team performance are introduced into regression model 2; the two dimensions of knowledge hiding are taken as independent variables, and temporary team performance is taken as dependent variables to introduce into regression model 3. Then, whether the dependent variable will be affected by the independent variable and the control variable is verified. The analysis results are shown in Figures 9, 10:

Figure 10 displays that model 1 is a regression analysis of team performance for control variables, such as team basic information. The regression coefficients of control variables, such as establishment time, team size, communication, and team type on temporary team performance are 0.009 (*P* < 0.05), 0.065 (*P* < 0.05), 0.058 (*P* < 0.05), and 0.127 (*P* < 0.001), respectively. Meanwhile, better communication among team members and team types are favorable to improving the work efficiency of the temporary team, and the establishment time and size of the team will not affect the work efficiency of the temporary team; Model 2 is a regression analysis of team performance based on independent variable knowledge hiding. Obviously, R^2^ is 0.654, after regulation, its value becomes 0.636, and the regression coefficient of model 2 is −0.69, indicating that knowledge hiding will reduce the work efficiency of temporary teams. Thus, hypothesis H1 is true; Model 3 is a regression analysis of team performance for two different categories of knowledge hiding. Apparently, R^2^ is 0.659, which becomes 0.637 after regulation. The regression coefficients of active hiding and passive hiding on temporary team performance are −0.452 and −0.254, respectively (*P* < 0.01), indicating that both active knowledge hiding and passive knowledge hiding are unfavorable to the improvement of temporary team performance. Hence, hypotheses H1a and H1B are true.

(2) Analysis of the mediating role of interpersonal mistrust

**FIGURE 11:**
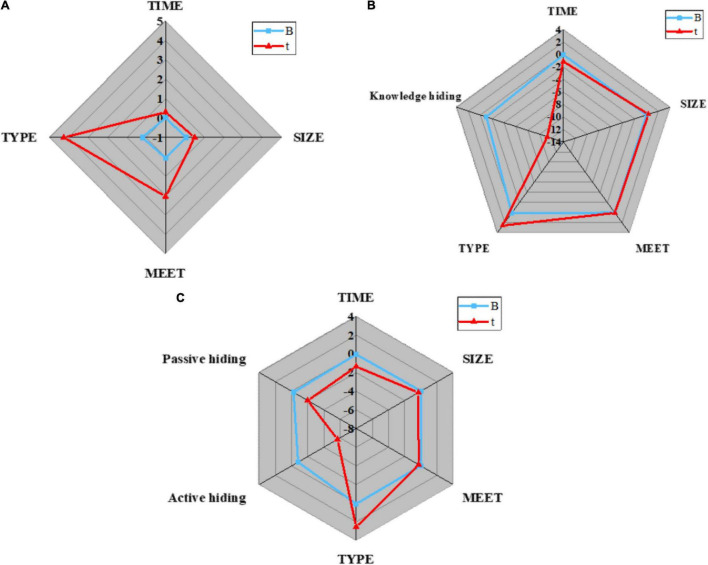
Regression analysis results of knowledge hiding on interpersonal distrust in different models. **(A)** Regression model 1; **(B)** Regression model 2; **(C)** Regression model 3.

**FIGURE 12:**
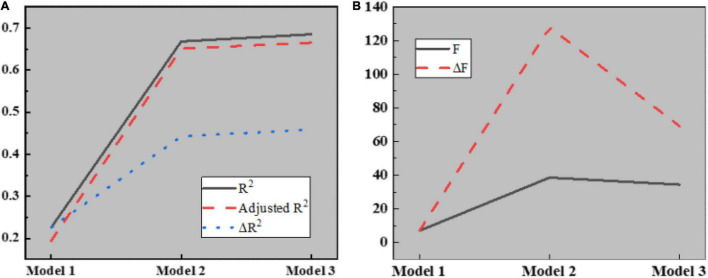
Parameters in regression analysis. **(A)** Parameters of R^2^; **(B)** Parameters of F.

Here, the causal step method is used to analyze the mediating role of interpersonal mistrust. The relationship between control variables, such as team basic information and interpersonal mistrust is introduced into regression model 1, the independent variable knowledge hiding and dependent variable interpersonal mistrust are introduced into regression model 2, and active hiding and passive hiding are taken as independent variables. Interpersonal distrust is introduced into regression model 3 as a dependent variable to test the regression relationship between knowledge hiding and interpersonal distrust. The results are shown in Figures 11, 12:

Figure 12 illustrates that model 1 is a regression analysis of interpersonal distrust by four control variables, such as the basic information of the team. Apparently, R^2^ is 0.226, which becomes 0.194 after regulation. The regression coefficients of control variables, such as team establishment time, team size, communication, and team type on interpersonal distrust are 0.017 (*P* < 0.05), 0.053 (*P* < 0.05), 0.069 (*P* < 0.05), and 0.195 (*P* < 0.001), respectively. Meanwhile, better communication and team type are favorable to improving the membership trust, while the establishment time and team size will not affect interpersonal relations; Model 2 is a regression analysis of the effect of knowledge hiding on interpersonal mistrust. Obviously, R^2^ is 0.668, and after regulation, its value becomes 0.651. The regression coefficient of model 2 is −0.808 (*P* < 0.001), indicating that knowledge hiding will enhance interpersonal mistrust; Model 3 is a regression analysis of interpersonal distrust for two different categories of knowledge hiding. Clearly, R^2^ is 0.685, which becomes 0.665 after regulation. The regression coefficients of active hiding and passive hiding on temporary team performance are −0.808 and −0.245, respectively (*P* < 0.001), indicating that both active knowledge hiding and passive knowledge hiding will aggravate interpersonal mistrust.

**FIGURE 13:**
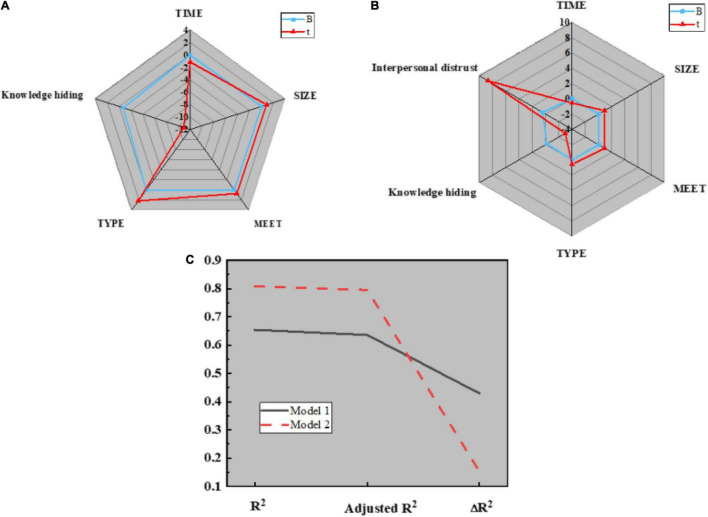
Regression analysis of the mediating effect of interpersonal mistrust on knowledge hiding and temporary team performance. **(A)** Regression model 1; **(B)** Regression model 2; **(C)** Parameter values.

**FIGURE 14:**
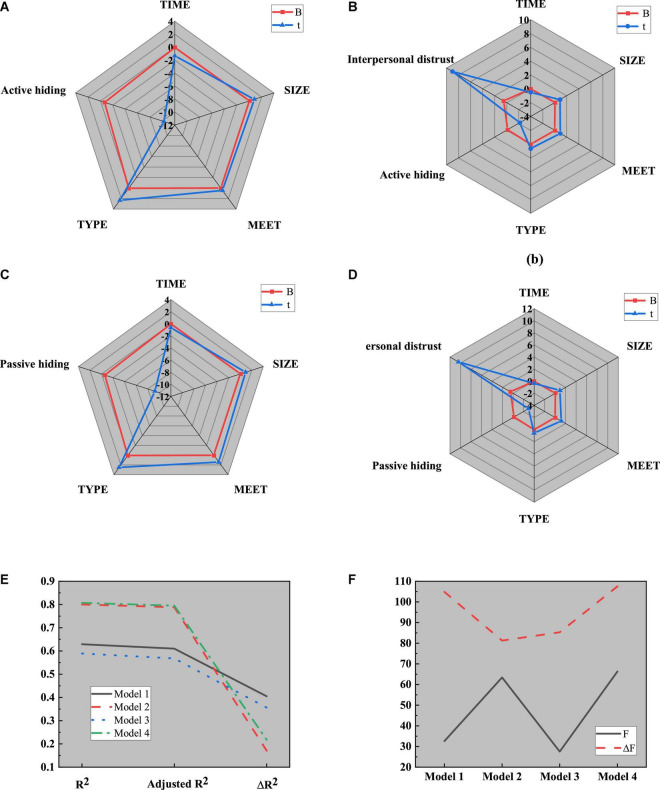
Regression analysis of the mediating role of interpersonal mistrust between active hiding, passive hiding, and temporary team performance. **(A)** Regression model 3; **(B)** Regression model 4; **(C)** Regression model 5; **(D)** Regression model 6; **(E)** Parameters of R^2^; **(F)** Parameters of F.

To sum up, knowledge hiding will worsen interpersonal trust. Therefore, knowledge hiding is taken as the independent variable, temporary team performance is taken as the dependent variable into regression model 1, knowledge hiding and mediating variable interpersonal distrust are put into regression model 2, and active hiding, passive hiding, and temporary team performance are put into regression models 3 and 5, respectively. Mediating variables are added to models 3 and 5, and interpersonal distrust is analyzed in regression models 4 and 6, respectively. The results are shown in Figures 13, 14:

Figure 14 implies that when the regression analysis is conducted on knowledge hiding, interpersonal distrust, and temporary team performance, R^2^ increases, and the regression coefficient between knowledge hiding and temporary team performance is significant at the level of 0.01, indicating that interpersonal distrust plays a mediating role between knowledge hiding and temporary team performance. Thus, hypothesis H2 is true; Comparative analysis of model 3 and model 4 tells that R^2^ increases, and the regression coefficient between active hiding and temporary team performance is significant at the level of 0.05, indicating that interpersonal distrust plays a mediating role between active hiding and temporary team performance. Thus, hypothesis H2a is true; The regression coefficient between passive hiding and temporary team performance is significant at the level of 0.01, indicating that interpersonal distrust plays a mediating role between passive hiding and temporary team performance, so hypothesis H2b is true.

(3) Analysis on the regulatory effect of control atmosphere and performance atmosphere

**FIGURE 15:**
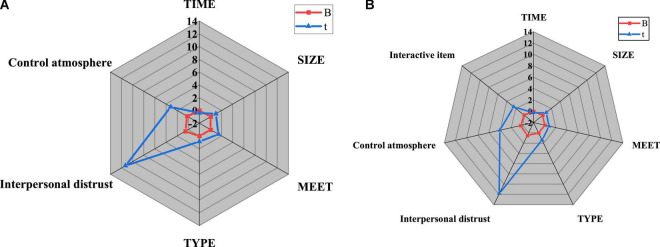
Mediating effect of control atmosphere on interpersonal mistrust and temporary team performance. **(A)** Regression model 1; **(B)** Regression model 2.

The role of control atmosphere between interpersonal mistrust and temporary team performance is studied, and the four variables involved in the team level, control atmosphere, temporary team performance, and interpersonal mistrust are introduced into regression model 1. The four variables involved in the team level, temporary team performance, the atmosphere control, and its interaction with interpersonal distrust are analyzed in regression model 2. The research results are shown in Figure 15:

Figure 15 concludes that model 1 is a regression analysis of control atmosphere, interpersonal distrust, and temporary team performance. The adjusted R^2^ is as high as 0.728. The regression coefficients of control atmosphere and interpersonal distrust on temporary team performance are 0.507 (*P* < 0.001) and 0.206 (*P* < 0.01), respectively. Evidently, interpersonal distrust will lead to low team efficiency, and the enhancement of control atmosphere is favorable to improving team performance; Model 2 is a regression analysis of the interaction item between interpersonal distrust and control atmosphere. The adjusted R^2^ is 0.808, and the regression coefficient of the interaction item on temporary team performance is 0.135 (*P* < 0.05), indicating that atmosphere control is favorable to the enhancement of membership trust and work efficiency. Thus, hypothesis H3 is true.

**FIGURE 16:**
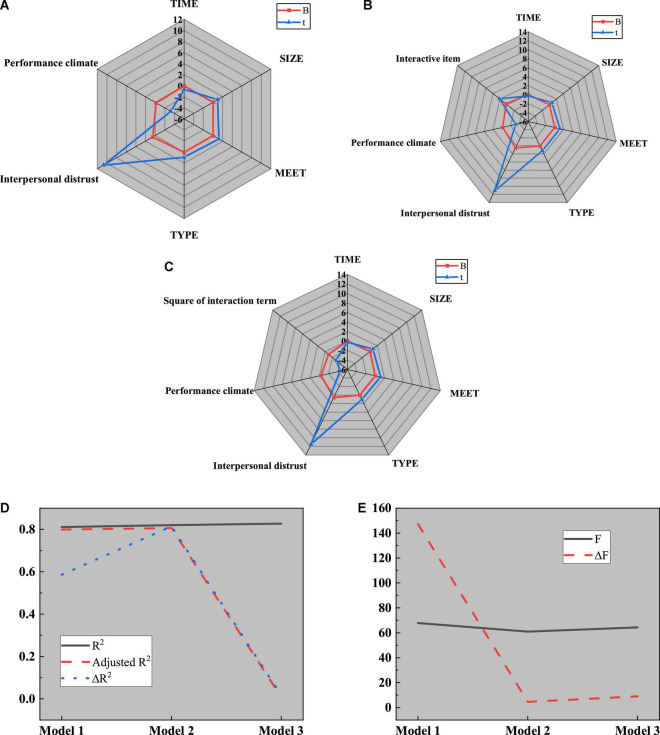
Test of the mediating effect of performance atmosphere on interpersonal mistrust and temporary team performance. **(A)** Regression model 1; **(B)** Regression model 2; **(C)** Regression model 3; **(D)** Parameters of R^2^; **(E)** Parameters of F.

Next, the role of performance atmosphere between interpersonal mistrust and temporary team performance is studied. The four variables involved in the team level, performance atmosphere, and interpersonal mistrust are introduced into regression model 1, and the performance atmosphere and its interaction with interpersonal mistrust are introduced into regression model 2. Meanwhile, the square of the interaction term composed of performance atmosphere and interpersonal distrust is introduced into regression model 3 for analysis. The research results are shown in Figure 16:

Figure 16 reveals that model 1 is a regression analysis of performance atmosphere, interpersonal distrust, and temporary team performance. The adjusted R^2^ is 0.799, and the regression coefficients of performance atmosphere and interpersonal distrust on temporary team performance are −0.186 (*P* < 0.01) and 0.496 (*P* < 0.001), respectively. Further analysis proves that interpersonal distrust and performance atmosphere will lead to low teamwork efficiency; The interaction term between interpersonal mistrust and performance atmosphere is added in model 2 to study the relationship between interpersonal mistrust and temporary team performance. The regression coefficient obtained is 0.293 (*P* > 0.05). Apparently, the performance atmosphere will not affect the relationship between interpersonal mistrust and temporary team performance. The interaction term between the square of performance atmosphere and interpersonal distrust is added to the regression model to test whether there is a U-shaped regulation. The regression coefficient obtained from the analysis is −0.959 (*P* < 0.01), indicating that there is an inverted U-shaped regulation. Thus, hypothesis H4 is true.

### Theoretical Implications

In summary, knowledge hiding will lead to low teamwork efficiency, interpersonal distrust plays a mediating role between knowledge hiding and temporary team performance, and the incentive atmosphere is favorable to improving membership trust and teamwork efficiency. Based on the previous relevant literature, different dimensions of knowledge hiding have different effects on team performance, but in the temporary team, both dimensions of knowledge hiding will lead to interpersonal mistrust. Our study has several implications to the literature on temporary team management.

First, this study provides empirical support for the important of the management of knowledge hiding in temporary team. Researchers have made great efforts to identify the consequences of knowledge sharing. In contrast, the negative effects of knowledge hiding are poorly understood in the context of temporary team. Our finding reveals that knowledge hiding has a significant negative impact on temporary team performance. It can be reasonably concluded that knowledge sharing does not bring better performance when the problem of knowledge hiding is not solved, which provides a new perspective for temporary team management.

Second, the direct impact of knowledge hiding on temporary team performance is only part of the story. To fully understand the role of knowledge hiding in temporary team, we use regression to analyze the mediating role of interpersonal distrust. It is found that interpersonal distrust plays a partially mediating role in the relationship between knowledge hiding and temporary team performance. This result suggests that there may be other variables that explain the potential mechanism between knowledge hiding and project team performance, and other potential mediating factors need to be explored from a more theoretical perspective.

Third, the findings of this paper provide a more subtle hint about the impact of incentive atmosphere in the temporary team. The management barriers of knowledge hiding caused by the temporary team-based work attributes deserve further study. Thus, we gain a more holistic view by focusing on the moderating effects of incentive atmosphere, namely team control atmosphere and performance atmosphere. The study found that team control atmosphere mitigated the negative effects of knowledge hiding on team learning. In addition, performance atmosphere is inverted U-shaped regulation between interpersonal distrust and temporary team performance. These findings can provide insights into managing knowledge hidden within temporary teams through effective work design.

### Management Enlightenment and Suggestions

Our findings have implications for knowledge management in temporary team.

(1) More communication opportunities should be provided for team members and encourage them to share themselves. It has been verified here that in the temporary team, the two dimensions of knowledge hiding can lead to interpersonal mistrust. In particular, a team culture should be established to minimize knowledge hiding, which pays more attention to personal development and team objectives. Under this cultural background, when team members can elevate their comprehensive quality by sharing, which can encourage their career advancement. Members who are willing to share can be rewarded materially and spiritually, as well as with honors. Large sharing meetings and expert symposiums can also be held regularly to improve the membership professionalism while reducing knowledge hiding.

(2) Passive hiding should be and cultivate a sense of trust between people. A large number of literatures show that passive hiding can have a great positive impact on the team and improve the work efficiency of team members. However, because the research object is temporary team, its stability is poor and it is sensitive to knowledge hiding, passive hiding can cause members’ resistance. Therefore, in the early stage of team establishment, more collective activities should be organized within the team to deepen the understanding among team members.

(3) Establish a more harmonious team atmosphere. A learning atmosphere should be formed within the team. Members should be encouraged to conduct vertical comparison of self-ability and cultivate collective consciousness. When creating a competitive atmosphere, pay attention to scale and encourage the improvement of personal performance, but not over exaggeration. At the same time, promote the formation of a community of destiny between individuals and teams, and resolutely resist the behavior of knowledge hiding for personal development.

Therefore, in team building, membership communication and knowledge sharing should be strengthened, and the knowledge hiding should be minimized, thereby building a good team cooperation atmosphere.

## Conclusion

This paper takes the knowledge-based temporary team as the research object, studies the relationship between knowledge hiding, interpersonal mistrust and temporary team performance, establishes relevant theoretical models, analyzes the basic information at the team level, and explores the mediating role of interpersonal mistrust. The conclusions are as follows:

(1) Knowledge hiding will lead to the decrease of interpersonal trust, which will have a negative impact on the work efficiency of the team, and the two dimensions of knowledge hiding, active hiding and passive hiding, are not favorable to the establishment of trust among team members;

(2) Interpersonal mistrust plays a mediating role between knowledge hiding and temporary team performance, which shows that knowledge hiding can affect the temporary performance of the team by affecting interpersonal relationships negatively;

(3) The control atmosphere is favorable to the establishment of a good team cooperation atmosphere, and the performance atmosphere shows an inverted “U” regulation. It means that a small degree of performance climate will motivate team members to improve their abilities to accomplish team tasks, in which case the performance climate positively regulates interpersonal trust relationships and interim team performance, while an overly competitive climate will negatively affect interim team performance.

## Data Availability Statement

The raw data supporting the conclusions of this article will be made available by the authors, without undue reservation.

## Ethics Statement

The studies involving human participants were reviewed and approved by the Wenzhou University Ethics Committee. The patients/participants provided their written informed consent to participate in this study. Written informed consent was obtained from the individual(s) for the publication of any potentially identifiable images or data included in this article.

## Author Contributions

All authors listed have made a substantial, direct, and intellectual contribution to the work, and approved it for publication.

## Conflict of Interest

The authors declare that the research was conducted in the absence of any commercial or financial relationships that could be construed as a potential conflict of interest.

## Publisher’s Note

All claims expressed in this article are solely those of the authors and do not necessarily represent those of their affiliated organizations, or those of the publisher, the editors and the reviewers. Any product that may be evaluated in this article, or claim that may be made by its manufacturer, is not guaranteed or endorsed by the publisher.
